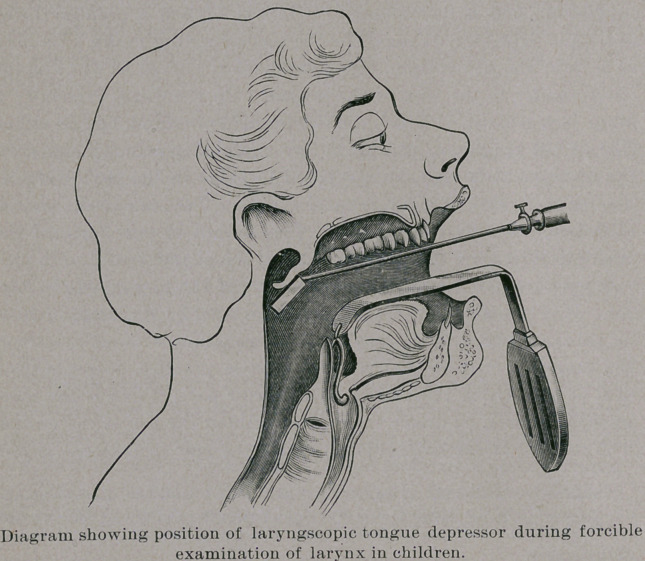# Forced Examination of the Larynx in Children

**Published:** 1899-04

**Authors:** 


### Abstracts and Selections.

### Forced Examination of the Larynx in Children.

It is sometimes extremely desirable to have a chance to make a detailed laryngoscopic examination of young children. One is apt to hesitate, however, to employ general anæsthesia, and parents will object to anæsthetics unless some operative procedure is intended at the same time. Besides, laryngoscopic examination under an anæsthetic is usually unsatisfactory. For a physician who does special work on the throat some method of accomplishing this purpose of satisfactory laryngoscopic examination of children is absolutely necessary.

In the forthcoming number of "*Progressive Medicine*,"* the new quarterly review of current medical progress, Dr. A. D. Blackader, of Montreal, will describe two novel methods. The first is Escat's suggestion, and is instrumental. He has devised a peculiar form of tongue depressor, as shown by the accompanying figure. As may be seen in the illustration, the instrument is curved so as to adapt itself exactly to the base of the tongue. On the distal extremity a blunt fork is fixed, of which the two branches descend, one on either side of the epiglottis, ending in two rounded points which, when the instrument is used, are supposed to lodge in the pyriform sinuses on each side of the laryngeal orifice. The instrument serves, therefore, not only to control the tongue, but to pull forward the rima glottidis from the posterior wall of the pharynx, and so to provide good conditions for the employment of the laryngoscopic mirror. It is probable that on the principles used by Kirstein, in what he calls autoscopy, i. e., laryngeal examination without a mirror, the examiner will be enabled with a little practice, to see a good deal of the larynx (especially its posterior part, which is the more important one), by direct vision, and without the use of the mirror. The method of the manipulations with the new instrument is well

---

*Progressive Medicine. A quarterly digest of new methods, discoveries and improvements in the Medical and Surgical Sciences. Edited by Hobart Amary Hare, M. D. Volume 1, March, 1899. Lea Brothers & Co., New York and Philadelphia.

illustrated in a diagram presented. In the second diagram the position of the instrument in the throat is well shown. It will, as a rule, be necessary, even with the instrument, to have the movements of the child restrained by a sheet rolled around its arms and legs in

Escat's laryngoscopic tongue depressor.

Diagram showing position of laryngscopic tongue depressor during forcible examination of larynx in children.

the usual way, and to have it carefully held on the knees of an assistant, but with this the examination of the larynx can be made much more satisfactorily than with the ordinary tongue depressor.

A simple method for the examination of young children is also given in the same number of *"Progressive Medicine,"* which seems extremely practical and well worth noting. It was demonstrated by Lack, at a meeting of the Laryngological Society of London, about a year ago. The advantage of this second method is that no special instruments are required and no force is employed. It is described by Dr. Blackader as follows: "The infant is placed in the usual position for laryngoscopy, the index finger of the left hand is passed well into the mouth, and the terminal phalanx hooked around the hyoid bone, which is pulled forward. The rest of the finger acts as a tongue depressor, the knuckle as a gag, while the left thumb under the chin serves to steady the head. With the use of a small mirror the larynx can now be easily seen. The method causes no pain, and requires no anæsthetic, while the younger the infant the less is the resistance and the easier the examination." These manipulations certainly commend themselves by their ease and simplicity, and it would seem that the method deserves thorough trial that its merits may be tested in practical use.

## Figures and Tables

**Figure f1:**
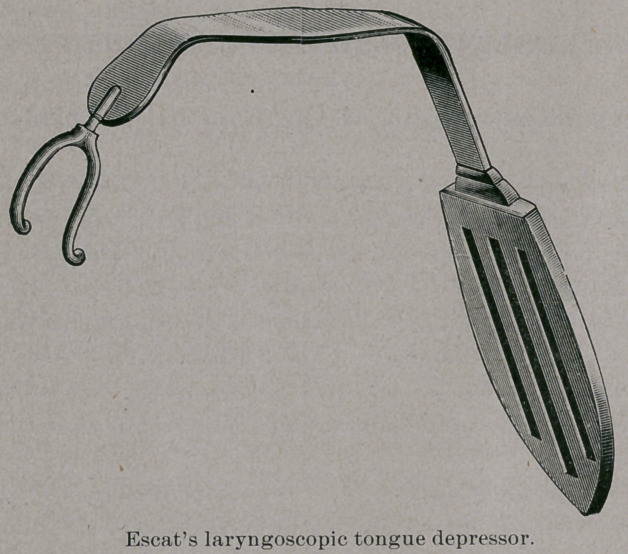


**Figure f2:**